# A Card of a “Windy City” Specialist

**Published:** 1898-06

**Authors:** 


A CARD OF A "WINDY CITY" SPECIALIST.

J— R— Veterinary Surgeon and Horse Hospital Infirmary. All Diseases of the Horse Successfully Treated Or No Pay.

I will give a written guarantee that a horse will never go lame on my shoeing. Moreover, I will cure by this discovery or secret all the following-named infirmities of the horse's feet, or no pay, by simply restoring the hoofs to their normal form, natural size, leveling, simitrising, and balancing the foundation of the horse, the *padal bone*, so as to give the indispensable right position of joints to prevent and cure Spavin, Ringbone, Splints, Enlargements of the Heels, Corns, Stringhalt, Knuckling, Curbs, Sprains of Tendons, Windgalls, Acute Liminitis, Founder, Knee-sprung, Contraction, all Crack-Hoof, Toroughpin, navicular Disease, Cutting, Overreaching, Interfering. I will send an ambulance for horses that are too sick to walk. Telephone —.

HORSE-SHOER.

---

Durland's Riding Academy, in New York, has just closed one of the most successful years in its history. The receipts exceed those of 1897 by $20,000. The number of saddle-horses in demand is greater than for many years, and higher quality is demanded. The bicycle has been no small factor in creating this changed condition by teaching thousands the pleasure and enjoyment of outdoor sport. Surely, blessings come forth from surprising sources.

